# (2-Amino­phen­yl)methanol

**DOI:** 10.1107/S1600536811053657

**Published:** 2011-12-21

**Authors:** Caitlin F. Zipp, Manuel A. Fernandes, Helder M. Marques, Joseph P. Michael

**Affiliations:** a Molecular Sciences Institute, School of Chemistry, University of the Witwatersrand, PO Wits 2050, Johannesburg, South Africa

## Abstract

The crystal strucure of the title compound, C_7_H_9_NO, displays N—H⋯O hydrogen bonds which link mol­ecules related by translation along the *b* axis, and O—H⋯N and further N—H⋯O hydrogen bonds which link mol­ecules related by the 2_1_ screw axis along the *c* axis. The resulting combination is a hydrogen-bonded layer of mol­ecules parallel to (011).

## Related literature

For the use of amines in the pharmaceutical industry, see: Morissette *et al.* (2004 ▶). For the use of amines in crystal engineering, see: Bernstein *et al.* (1999 ▶). For hydrogen-bond motifs, see: Bernstein *et al.* (1995 ▶); Etter *et al.* (1990 ▶).
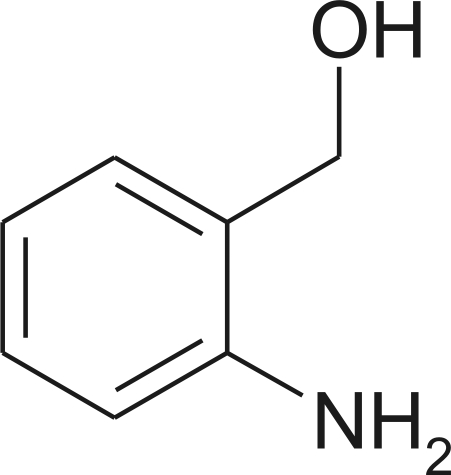


## Experimental

### 

#### Crystal data

C_7_H_9_NO
*M* *_r_* = 123.15
Orthorhombic, 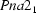

*a* = 22.6222 (9) Å
*b* = 6.0675 (2) Å
*c* = 4.7005 (2) Å
*V* = 645.19 (4) Å^3^
*Z* = 4
Mo *K*α radiation
μ = 0.09 mm^−1^
*T* = 173 K
0.46 × 0.20 × 0.07 mm

#### Data collection

Bruker APEXII CCD diffractometer
4682 measured reflections
715 independent reflections
681 reflections with *I* > 2σ(*I*)
*R*_int_ = 0.078

#### Refinement

*R*[*F* ^2^ > 2σ(*F* ^2^)] = 0.030
*wR*(*F* ^2^) = 0.075
*S* = 1.09
715 reflections
82 parameters
1 restraint
H-atom parameters constrained
Δρ_max_ = 0.12 e Å^−3^
Δρ_min_ = −0.13 e Å^−3^

### 

Data collection: *APEX2* (Bruker, 2005 ▶); cell refinement: *SAINT* (Bruker, 2005 ▶); data reduction: *SAINT*; program(s) used to solve structure: *SHELXS97* (Sheldrick, 2008 ▶); program(s) used to refine structure: *SHELXL97* (Sheldrick, 2008 ▶); molecular graphics: *ORTEP-3 for Windows* (Farrugia, 1997 ▶) and *SCHAKAL99* (Keller, 1999 ▶); software used to prepare material for publication: *WinGX* (Farrugia, 1999 ▶) and *PLATON* (Spek, 2009 ▶).

## Supplementary Material

Crystal structure: contains datablock(s) global, I. DOI: 10.1107/S1600536811053657/fj2480sup1.cif

Structure factors: contains datablock(s) I. DOI: 10.1107/S1600536811053657/fj2480Isup2.hkl

Supplementary material file. DOI: 10.1107/S1600536811053657/fj2480Isup3.cml

Additional supplementary materials:  crystallographic information; 3D view; checkCIF report

## Figures and Tables

**Table 1:** Hydrogen-bond geometry (Å, °)

*D*—H⋯*A*	*D*—H	H⋯*A*	*D*⋯*A*	*D*—H⋯*A*
O1—H1⋯N1^i^	0.85	1.94	2.791 (2)	172
N1—H1*B*⋯O1^i^	0.91	2.28	3.135 (2)	156
N1—H1*A*⋯O1^ii^	0.87	2.19	3.0585 (17)	175

Symmetry codes: (i) 
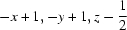
; (ii) 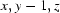.

**Fig. 1.**
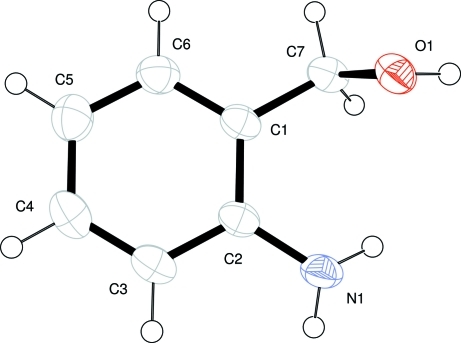
The molecular structure of (I), showing the atomic numbering scheme. Displacement ellipsoids are drawn at the 50% probability level.

**Fig. 2.**
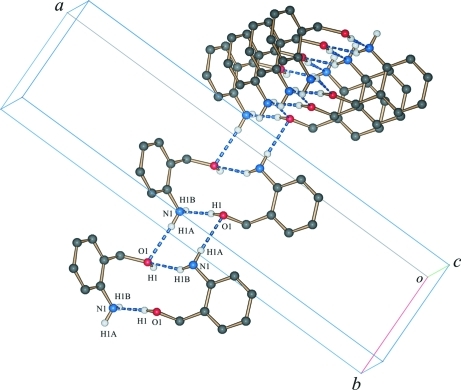
Diagram showing the intermolecular N—H···O and O—H···N hydrogen bonding network in the structure of (I). Molecules related by translation along the b axis are held together by the N1—H1A···O1 hydrogen bond. In addition, molecules related by the 2 fold screw axis along c are held together by the O1—H1···N1 and N1—H1B···O1 hydrogen bonds which appear as a stack of molecules when viewed down the c axis.

## supplementary crystallographic information

### Comment

Amines play an important role in various areas of chemistry. Amines are used as precursors to amide and peptide functional groups in organic chemistry. The acid-base properties of amines are important in the synthesis of salts. These properties, as well as their hydrogen bonding capabilities, make amines an important functionality in the pharmaceutical industry (Morissette *et al.*, 2004). The hydrogen bonding capabilities of amines also make them an important component of the crystal engineer's arsenal (Bernstein *et al.*, 1999).

The title compound (I) is capable of forming hydrogen bonds through the alcohol and amine groups (Fig. 1). In this structure, molecules related by translation along the *b* axis are linked by the N1—H1A···O1 hydrogen bond to form a C6 chain (Etter *et al.*, 1990; Bernstein *et al.*, 1995) along the *b* axis. In addition, molecules related by the 2 fold screw axis along c, are held together by the O1—H1···N1 hydrogen bond and the N1—H1B···O1 to form a chain of molecules which appear as a stack of molecules when viewed down the *c* axis (Fig. 2). The combination of these two hydrogen bonded chains results in a hydrogen bonded layer of molecules parallel to (011).

### Experimental

The title compound was purchased from Sigma Aldrich and was recrystallized from dichloromethane and hexane (1:1) to yield colourless needles.

### Refinement

With the exception of those involved in hydrogen bonding, all H atoms were first located in the difference Fourier map and then positioned geometrically, and allowed to ride on their parent atoms. Hydrogen bond lengths were set as follows for C—H = 0.95 Å (CH) or 0.99 Å (CH_2_). Hydrogen atoms involved in hydrogen bonding (N—H and O—H) were located in the difference Fourier map and then allowed to ride on their parent atoms with unmodified N—H and O—H distances. Isotropic displacement parameters for the H atoms were set as follows: 1.2 times *U*_eq_ of the parent atom for C and N, and 1.5 times *U*_eq_ of the parent atom for O. Though the molecule crystallizes in a polar space group it was not possible to determine the absolute conformation of the crystal. As a consequence all Friedel pairs were merged during the final refinements with a *SHELXL97* MERG 4 instruction.

### Crystal data

**Table:**

C_7_H_9_NO	*F*(000) = 264
*M**_r_* = 123.15	*D*_x_ = 1.268 Mg m^−^^3^
Orthorhombic, *P**n**a*2_1_	Mo *K*α radiation, λ = 0.71073 Å
Hall symbol: P 2c -2n	Cell parameters from 3105 reflections
*a* = 22.6222 (9) Å	θ = 3.5–28.3°
*b* = 6.0675 (2) Å	µ = 0.09 mm^−^^1^
*c* = 4.7005 (2) Å	*T* = 173 K
*V* = 645.19 (4) Å^3^	Needle, colourless
*Z* = 4	0.46 × 0.20 × 0.07 mm

### Data collection

**Table:**

Bruker APEXII CCD diffractometer	681 reflections with *I* > 2σ(*I*)
Radiation source: fine-focus sealed tube	*R*_int_ = 0.078
graphite	θ_max_ = 26.0°, θ_min_ = 1.8°
φ and ω scans	*h* = −27→27
4682 measured reflections	*k* = −7→6
715 independent reflections	*l* = −5→5

### Refinement

**Table:**

Refinement on *F*^2^	Primary atom site location: structure-invariant direct methods
Least-squares matrix: full	Secondary atom site location: difference Fourier map
*R*[*F*^2^ > 2σ(*F*^2^)] = 0.030	Hydrogen site location: inferred from neighbouring sites
*wR*(*F*^2^) = 0.075	H-atom parameters constrained
*S* = 1.09	*w* = 1/[σ^2^(*F*_o_^2^) + (0.0244*P*)^2^ + 0.1192*P*] where *P* = (*F*_o_^2^ + 2*F*_c_^2^)/3
715 reflections	(Δ/σ)_max_ < 0.001
82 parameters	Δρ_max_ = 0.12 e Å^−^^3^
1 restraint	Δρ_min_ = −0.13 e Å^−^^3^

### Special details

**Table:**

Geometry. All e.s.d.'s (except the e.s.d. in the dihedral angle between two l.s. planes) are estimated using the full covariance matrix. The cell e.s.d.'s are taken into account individually in the estimation of e.s.d.'s in distances, angles and torsion angles; correlations between e.s.d.'s in cell parameters are only used when they are defined by crystal symmetry. An approximate (isotropic) treatment of cell e.s.d.'s is used for estimating e.s.d.'s involving l.s. planes.
Refinement. Refinement of *F*^2^ against ALL reflections. The weighted *R*-factor *wR* and goodness of fit *S* are based on *F*^2^, conventional *R*-factors *R* are based on *F*, with *F* set to zero for negative *F*^2^. The threshold expression of *F*^2^ > σ(*F*^2^) is used only for calculating *R*-factors(gt) *etc*. and is not relevant to the choice of reflections for refinement. *R*-factors based on *F*^2^ are statistically about twice as large as those based on *F*, and *R*- factors based on ALL data will be even larger.

### Fractional atomic coordinates and isotropic or equivalent isotropic displacement parameters (Å^2^)

**Table:**

	*x*	*y*	*z*	*U*_iso_*/*U*_eq_	
C1	0.38086 (8)	0.4709 (3)	0.5647 (4)	0.0316 (4)	
C2	0.40551 (8)	0.2625 (3)	0.6243 (5)	0.0306 (4)	
C3	0.37720 (9)	0.1240 (3)	0.8170 (5)	0.0378 (5)	
H3	0.3938	−0.0161	0.8583	0.045*	
C4	0.32549 (9)	0.1866 (3)	0.9489 (6)	0.0443 (5)	
H4	0.3067	0.0889	1.0783	0.053*	
C5	0.30074 (9)	0.3917 (4)	0.8937 (6)	0.0450 (5)	
H5	0.2653	0.4364	0.9855	0.054*	
C6	0.32908 (9)	0.5302 (3)	0.7009 (5)	0.0383 (5)	
H6	0.3122	0.6704	0.6615	0.046*	
C7	0.40968 (8)	0.6224 (3)	0.3546 (5)	0.0348 (5)	
H7A	0.4151	0.5445	0.1714	0.042*	
H7B	0.3839	0.7515	0.3207	0.042*	
N1	0.45972 (7)	0.1975 (2)	0.5061 (4)	0.0343 (4)	
H1A	0.4637	0.0557	0.4904	0.041*	
H1B	0.4735	0.2644	0.3450	0.041*	
O1	0.46612 (5)	0.69530 (18)	0.4600 (3)	0.0350 (4)	
H1	0.4872	0.7176	0.3123	0.053*	

### Atomic displacement parameters (Å^2^)

**Table:**

	*U*^11^	*U*^22^	*U*^33^	*U*^12^	*U*^13^	*U*^23^
C1	0.0345 (9)	0.0258 (8)	0.0344 (10)	−0.0039 (7)	−0.0059 (9)	0.0007 (8)
C2	0.0357 (9)	0.0243 (8)	0.0319 (9)	−0.0036 (7)	−0.0055 (10)	−0.0008 (8)
C3	0.0440 (11)	0.0279 (9)	0.0415 (12)	−0.0051 (8)	−0.0051 (10)	0.0048 (10)
C4	0.0448 (11)	0.0428 (11)	0.0451 (12)	−0.0116 (9)	0.0010 (11)	0.0089 (11)
C5	0.0360 (11)	0.0488 (12)	0.0501 (13)	−0.0034 (9)	0.0034 (11)	0.0022 (10)
C6	0.0356 (10)	0.0335 (10)	0.0458 (13)	0.0011 (8)	−0.0041 (10)	0.0021 (9)
C7	0.0386 (10)	0.0281 (9)	0.0377 (11)	−0.0005 (8)	−0.0042 (9)	0.0035 (9)
N1	0.0424 (9)	0.0216 (7)	0.0387 (10)	0.0011 (6)	0.0016 (8)	0.0001 (7)
O1	0.0387 (7)	0.0284 (6)	0.0380 (8)	−0.0055 (5)	0.0009 (7)	0.0009 (6)

### Geometric parameters (Å, °)

**Table:**

C1—C6	1.383 (3)	C5—C6	1.393 (3)
C1—C2	1.410 (2)	C5—H5	0.9500
C1—C7	1.498 (3)	C6—H6	0.9500
C2—C3	1.392 (3)	C7—O1	1.439 (2)
C2—N1	1.403 (2)	C7—H7A	0.9900
C3—C4	1.377 (3)	C7—H7B	0.9900
C3—H3	0.9500	N1—H1A	0.8683
C4—C5	1.389 (3)	N1—H1B	0.9141
C4—H4	0.9500	O1—H1	0.8534
			
C6—C1—C2	118.46 (17)	C6—C5—H5	120.8
C6—C1—C7	120.94 (16)	C1—C6—C5	122.29 (18)
C2—C1—C7	120.59 (17)	C1—C6—H6	118.9
C3—C2—N1	119.38 (16)	C5—C6—H6	118.9
C3—C2—C1	119.25 (18)	O1—C7—C1	110.35 (17)
N1—C2—C1	121.26 (16)	O1—C7—H7A	109.6
C4—C3—C2	121.18 (18)	C1—C7—H7A	109.6
C4—C3—H3	119.4	O1—C7—H7B	109.6
C2—C3—H3	119.4	C1—C7—H7B	109.6
C3—C4—C5	120.3 (2)	H7A—C7—H7B	108.1
C3—C4—H4	119.8	C2—N1—H1A	113.8
C5—C4—H4	119.8	C2—N1—H1B	120.1
C4—C5—C6	118.5 (2)	H1A—N1—H1B	109.5
C4—C5—H5	120.8	C7—O1—H1	105.4
			
C6—C1—C2—C3	−0.1 (3)	C3—C4—C5—C6	0.6 (3)
C7—C1—C2—C3	−178.96 (19)	C2—C1—C6—C5	0.1 (3)
C6—C1—C2—N1	−176.24 (17)	C7—C1—C6—C5	179.0 (2)
C7—C1—C2—N1	4.9 (3)	C4—C5—C6—C1	−0.3 (3)
N1—C2—C3—C4	176.60 (19)	C6—C1—C7—O1	114.52 (18)
C1—C2—C3—C4	0.4 (3)	C2—C1—C7—O1	−66.6 (2)
C2—C3—C4—C5	−0.7 (3)		

### Hydrogen-bond geometry (Å, °)

**Table:**

*D*—H···*A*	*D*—H	H···*A*	*D*···*A*	*D*—H···*A*
O1—H1···N1^i^	0.85	1.94	2.791 (2)	172
N1—H1B···O1^i^	0.91	2.28	3.135 (2)	156
N1—H1A···O1^ii^	0.87	2.19	3.0585 (17)	175

Symmetry codes: (i) −*x*+1, −*y*+1, *z*−1/2; (ii) *x*, *y*−1, *z*.
